# Modeling and correction of image drift in dynamic shadowgraphy experiments

**DOI:** 10.1140/epje/s10189-024-00413-y

**Published:** 2024-04-08

**Authors:** Stefano Castellini, Matteo Brizioli, Cédric Giraudet, Marina Carpineti, Fabrizio Croccolo, Fabio Giavazzi, Alberto Vailati

**Affiliations:** 1 Dipartimento di Fisica“A. Pontremoli”, Università degli Studi di Milano, Milan, Italy; 2 Dipartimento di Biotecnologie Mediche e Medicina Traslazionale, Università degli Studi di Milano, Segrate, Italy; 3 LFCR UMR5150, E2S UPPA, CNRS, Universite de Pau et des Pays de l’Adour, Anglet, France

## Abstract

**Abstract:**

The study of phoretic transport phenomena under non-stationary conditions presents several challenges, mostly related to the stability of the experimental apparatus. This is particularly true when investigating with optical means the subtle temperature and concentration fluctuations that arise during diffusion processes, superimposed to the macroscopic state of the system. Under these conditions, the tenuous signal from fluctuations is easily altered by the presence of artifacts. Here, we address an experimental issue frequently reported in the investigation by means of dynamic shadowgraphy of the non-equilibrium fluctuations arising in liquid mixtures under non-stationary conditions, such as those arising after the imposition or removal of a thermal stress, where experiments show systematically the presence of a spurious contribution in the reconstructed structure function of the fluctuations, which depends quadratically from the time delay. We clarify the mechanisms responsible for this artifact, showing that it is caused by the imperfect alignment of the sample cell with respect to gravity, which couples the temporal evolution of the concentration profile within the sample with the optical signal collected by the shadowgraph diagnostics. We propose a data analysis protocol that enables disentangling the spurious contributions and the genuine dynamics of the fluctuations, which can be thus reliably reconstructed.

**Graphic Abstract:**

The imposition of a thermal gradient across a liquid mixture results in a time-dependent refractive index distribution. In the presence of a misalignment of the confining cell with respect to gravity, this leads to a deflection of the optical probe beam used to monitor concentration fluctuations within the sample in quantitative shadowgraphy experiments. If not properly accounted for, this effect can introduce a significant bias in the optical signal.
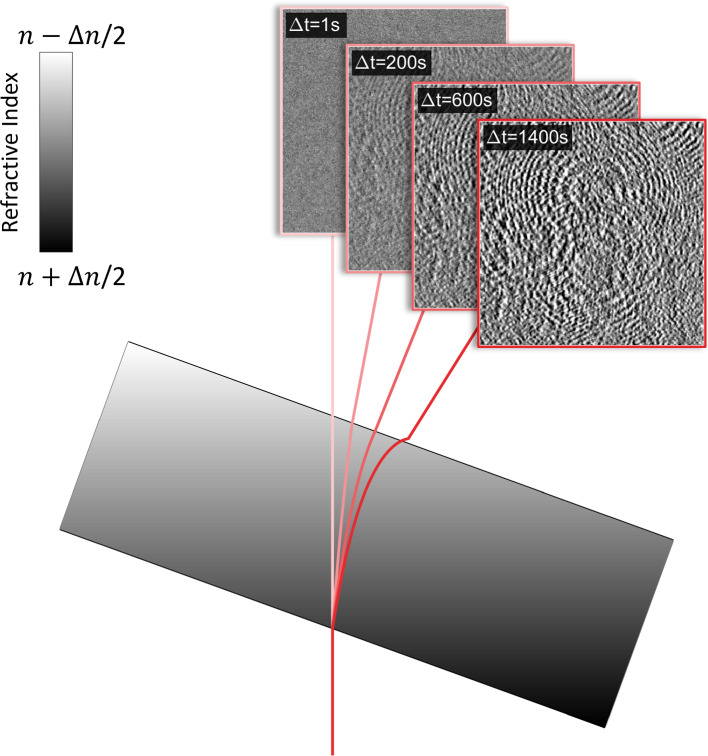

## Introduction

Light scattering methods represent a noninvasive approach for the characterization of the transport coefficients of simple and complex fluids, by exploiting the fact that the fluid undergoes continuously spontaneous temperature and concentration fluctuations determined by the thermal agitation of the molecules [1]. These fluctuations are always present in fluids, even under equilibrium conditions, in the absence of a macroscopic temperature or concentration gradient. According to Onsager regression hypothesis, equilibrium fluctuations relax by following the same equations of the macroscopic state. Therefore, the statistical characterization of fluctuations under equilibrium conditions allows investigating transport processes without the need to induce a macroscopic non-equilibrium process. This feature has made light scattering methods a very popular and powerful tool for the characterization of complex fluids.

During the last 25 years, it has been shown that macroscopic transport processes are accompanied by “giant” non-equilibrium temperature and concentration fluctuations, whose amplitude can be orders of magnitude larger than those of equilibrium fluctuations at small wave vectors [2, 3]. Indeed, in the case of mass diffusion processes, it has been shown that these fluctuations represent mass currents at the mesoscopic scale, whose overall contribution is the macroscopic Fick’s flux [4, 5]. Due to the fact that they occur in a fluid stratified in density, these fluctuations are strongly affected by the gravity force at small wave vector, preventing their diffusive relaxation [6–10]. Due to their large amplitude, non-equilibrium fluctuations represent a promising and powerful tool to characterize the transport properties of complex fluids [11–13], including phoretic coefficients of macromolecules in external fields, which cannot be determined under equilibrium conditions. In the past, a huge attention has been devoted to the theoretical and experimental investigation of fluctuations occurring either at equilibrium or during a macroscopic transport process under steady-state conditions [2]. In both these ideal configurations, the stationarity of the process allows the accumulation of a statistical sample as large as desired of the fluctuations, at the expense of a long measurement time. Therefore, using dynamic light scattering techniques it is possible to achieve a reliable characterization of the mean square amplitude and relaxation time of feeble temperature and concentration non-equilibrium fluctuations [14–16]. A limitation of traditional dynamic light scattering methods is that, to maximize the contrast of the signal, one needs to collect the intensity fluctuations from a single coherence area, thus strongly limiting the spatial extension of the volume of sample that can be investigated. Under these conditions, a statistical characterization of the fluctuations is possible only by performing long measurements, so that the system can explore all the accessible states. A further limitation of traditional dynamic light scattering methods is that the time correlation function of the scattered light is typically collected at a single wave vector at a time. Starting from the beginning of this century, several different optical techniques that allow overcoming the limitations of DLS have been developed [17]. These techniques include near-field scattering [18–20], quantitative dynamic shadowgraphy [10] and Schlieren [21, 22], and differential dynamic microscopy [23–25], which typically work by collecting on a matrix sensor the light scattered in the near field where it interferes with the main probe beam that illuminates the sample. A great advantage of these near-field methods is that, due to the interference with the main beam, the signal collected by the sensor is proportional to the scattered field rather than to the scattered intensity, a feature that determines a sensitivity much larger than that of traditional far-field scattering methods. The analysis of the signal involves the Fourier decomposition of the interference patterns collected in the near field and their processing to achieve a statistical characterization of the static and dynamic structure factors of the sample under investigation [17]. With respect to DLS, which requires a small scattering volume to achieve an optimal contrast of the signal, near-field methods work with arbitrarily large scattering volume with two remarkable advantages. The first one is that the scattered signal can be collected simultaneously at several different wave vectors. With the currently available sensors, whose resolution exceeds one megapixel, the number of wave vectors explored can be typically of the order of one million. The second advantage of these methods is that the large number of independent wave vectors explored allows to perform an ensemble average of different states of the system. In the case of ergodic systems, this ensemble average is equivalent to the time average performed in DLS, which requires however much longer measurement times. The significant advantages of near-field methods have made them increasingly popular in the scientific community. Near-field scattering has been profitably used to investigate colloidal aggregation induced by critical Casimir forces under microgravity conditions [26]. DDM has been applied widely to the investigation of biological systems [27–30], non-equilibrium fluctuations in colloidal suspensions [31], and near-critical binary mixtures [32] and will be used to investigate fluctuations in sedimenting colloidal suspensions under various gravitational conditions on the International Space Station within the framework of the “Sedimenting Colloids” project of the European Space Agency. Quantitative Shadowgraphy is the technique of choice to investigate non-equilibrium fluctuations induced by thermal gradients in single component liquids [33] and in multi-component liquid mixtures [11, 34, 35], where the temperature gradient induces a mass flow through the Soret effect. It has been profitably used to investigate non-equilibrium temperature fluctuations in CS$_2$ [36] and concentration fluctuations in a polymer solution [13, 37, 38] in the absence of gravity in the framework of the GRADFLEX project of the European Space Agency, which flew on FOTON M3 in 2007. All these near-field methods have proven their effectiveness for the characterization of fluctuations in equilibrium and non-equilibrium systems under stationary or quasi-stationary conditions in the presence of small gradients. These are the ideal conditions that allow a theoretical modeling of fluctuations by using linearized hydrodynamics [2, 39, 40]. Currently, the attention has been shifted to the investigation of non-equilibrium fluctuations in systems undergoing non-stationary diffusion processes [38, 41–43], because many natural and technological diffusion processes occur under these conditions that cannot be tackled easily with linearized hydrodynamics, unless an adiabatic approximation can be made to separate macroscopic and mesoscopic variables on the basis of their different evolution times [44]. On Earth, gravity determines a stabilization of non-equilibrium fluctuations at small wave vectors [6–8, 45] and the macroscopic and mesoscopic variables can be separated. In the absence of gravity, this separation is not possible, and the diffusion process involves simultaneously all the length scales from the microscopic to the macroscopic ones. For these reasons, the investigation of transient diffusion processes in the absence of gravity has a broad fundamental interest. Indeed, currently, several space projects of the European Space Agency are focused on the fundamental investigation of fluctuations during transient diffusion processes in complex fluids under various gravitational conditions, because the understanding of the stability of liquid mixtures in space is a strategic habilitating factor for space exploration [46, 47]. These projects include Giant Fluctuations (Neuf-Dix) and TechNES on the International Space Station (ISS) [48, 49], Sedimenting Colloids on the Flumias facility of the ISS, and NESTEX on the ISS and the Chinese Space Station. The facility developed for the Giant Fluctuations project is a sophisticated 2-colors shadowgraph diagnostics in combination with thermal gradient cells, with optical windows perpendicular to the gradient. The imposition of a thermal gradient to a multi-component liquid mixture determines a mass flow of its components and in turn the development of macroscopic concentration gradients that give rise to non-equilibrium concentration fluctuations.

The mutual alignment of the temperature gradient $\nabla T$ and gravitational acceleration $\textbf{g}$ strongly affects the stability of the sample during experiments. When the thermal gradient is perpendicular to the gravitational acceleration, the configuration is that of a thermogravitational column [50], and the sample is unstable against convection, which occurs in the presence of an arbitrarily small gradient. The case of interest for the investigation of non-equilibrium fluctuations is the stable configuration where the temperature gradient is parallel to the acceleration of gravity and the density profile inside the sample is stable [2]. In practice, attaining an experimental configuration where $\nabla T$ and $\textbf{g}$ are perfectly parallel is impossible, and the two will be in general aligned within an arbitrarily small tilt angle $\theta$.

In this work, we investigate the impact of such a small inevitable tilt on the investigation of non-equilibrium fluctuations by means of quantitative shadowgraphy. We show that the tilt does not affect measurements performed under steady-state conditions, but determines the presence of a spurious quadratic term that affects the long time behavior of the correlation function of non-equilibrium fluctuations during transient diffusion processes. The modeling of the propagation of the beam shows that the presence of a tilt gives rise to a component of the concentration gradient perpendicular to the beam, which determines its deflection. During a transient diffusion process, this deflection is time-dependent and gives rise to a gradual shift of the shadowgraph images collected by the sensor. The deflection of the beam occurs under generic conditions, both in the presence of a linear concentration profile, or of a nonlinear concentration profile characterized by a concentration dependence of the diffusion coefficient [43]. We develop a protocol for the analysis of the experimental results that allows decoupling this spurious contribution from the actual time correlation function of non-equilibrium fluctuations.

## Materials and methods

### Sample

All experiments presented in this paper are performed on a binary mixture of polystyrene polymer of molecular weight 9,100 g mol$^{-1}$ and toluene. The sample is prepared with a polystyrene concentration $c=2.0\%$ w/w. The same sample has been utilized in previous work in the area of non-equilibrium fluctuations, such as the GRADFLEX space mission [37], and its thermophysical properties are well characterized in the literature [51–53]. For reference, at $30^{\circ }$C, the solutal diffusion coefficient is $D_0=1.97 \cdot 10^{-6}$ cm$^2$/s, the kinematic viscosity $\nu =6.39 \cdot 10^{-3}$ cm$^2/$s, the Soret coefficient is $S_T=6.486\cdot 10^{-2}$ K$^{-1}$, the concentration optical contrast factor is $\frac{\text {d}n}{\text {d}c}|_{T,p}=8.951 \cdot 10^{-2}$ and, finally, the thermal conductivity is $\kappa =0.1309$ W/(m K).

### Experimental setup

**Fig. 1:**
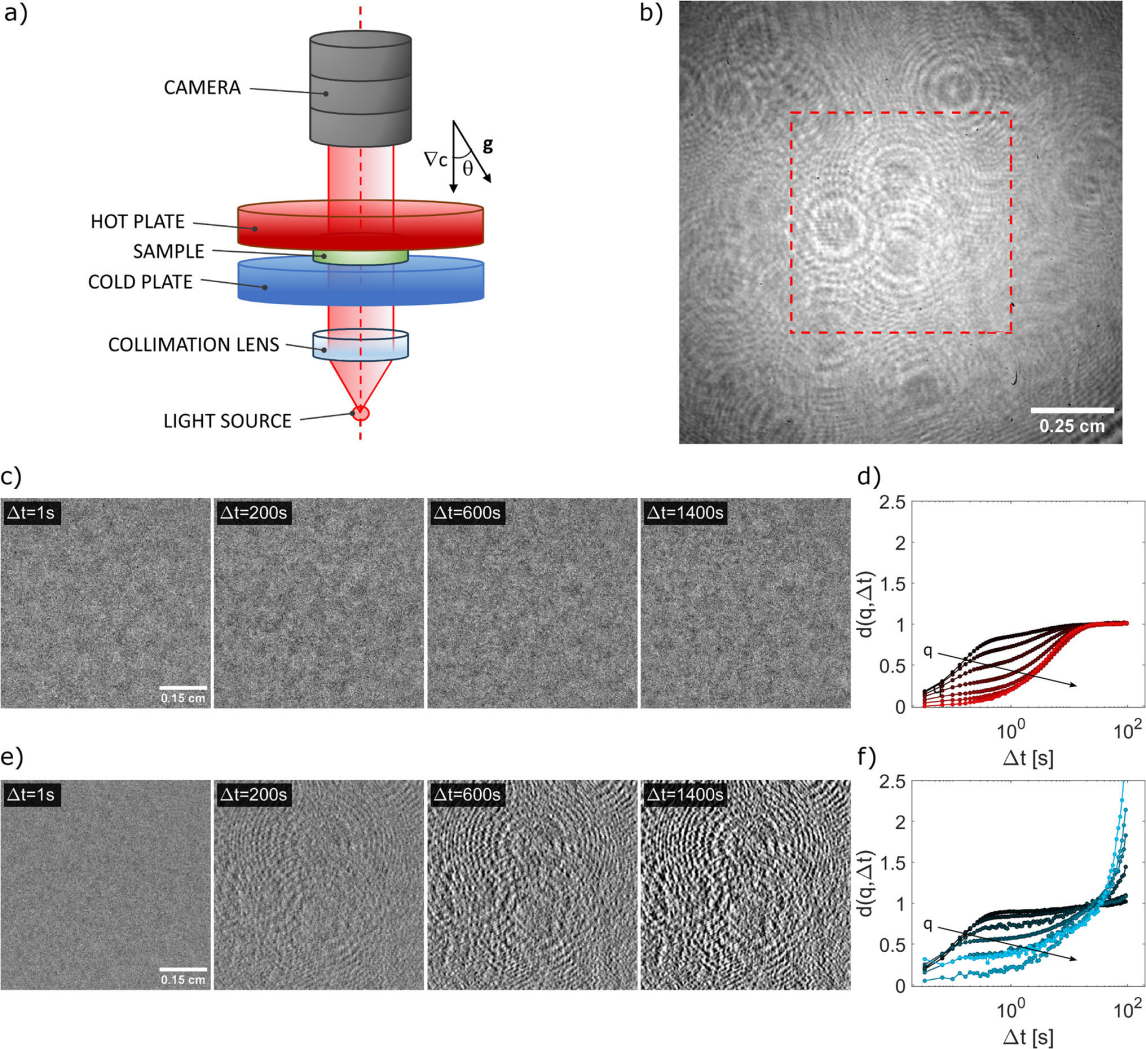
Dynamic shadowgraph experiment in stationary and non-stationary conditions: **a** Schematic representation of the experimental apparatus, including the thermal gradient cell and shadowgraph diagnostics. The distance between the sample cell and the camera sensor is $z=45$ cm. **b** Representative raw shadowgraph image. The red dashed square corresponds to the ROI shown in (**c**) and (**e**). **c** Sequence of images collected at different times in stationary conditions during a typical experiment, where a steady temperature difference $\varDelta T = 17$ K is imposed across the sample. The image collected at a reference time $t=0$ has been subtracted from all the other images. This enables efficiently removing the optical background and highlighting the contribution of thermal and solutal NEF to the image intensity. **d** Corresponding normalized image structure functions $d(q,\varDelta t)$ for different *q*-values in the range [0.72–3.2] $\cdot 10^2$ cm$^{-1}$ featuring two distinct decays corresponding to the relaxation of thermal and solutal NEF, respectively. **e** Sequence of images collected at different times in non-stationary conditions, after switching off, at time $t=0$ s, the thermal gradient. As a reference image, we consider the one collected at time $t=200$ s, which has been subtracted from all the other images. **f** Corresponding normalized image structure functions $d(q,\varDelta t)$ for different *q*-values in the range [0.72,3.2] $\cdot 10^2$ cm$^{-1}$, displaying a single decay (corresponding to the relaxation of solutal NEF) followed by a marked divergence for long time delays

The sample is confined laterally by an O-ring with inner diameter of 43 mm and sandwiched between two sapphire windows through which a temperature gradient is applied (Fig. 1a). The thickness of the sample is defined by the distance between the two sapphire windows, which is determined by five calibrated Delrin spacers with a thickness $h = 1.30$ mm. The temperature of the sapphire windows is controlled by two annular thermoelectric devices (TEDs), with an inner opening that provides optical access to a circular region with a diameter of 27 mm. One side of each TED is thermally coupled to a sapphire window by means of an aluminum ring, while the other side is in contact with an annular flange maintained at a constant temperature of $19.0\,^\circ$C by means of a water circulating thermostat. In this work, we consider both experiments performed under stationary and non-stationary NE conditions. In all cases, at the beginning of the experiment, the sample is in a stratified state, characterized by a steady linear concentration gradient generated by thermophoresis. This is obtained by imposing a steady temperature gradient across the sample for a suitably long time ($\sim$ 4000 s), heating from above, so that a stationary concentration gradient $\nabla c= S_T \,c(1-c)\, \nabla T$ gradually develops in time. Experiments under stationary conditions are performed maintaining the same temperature difference for their whole duration. Experiments under non-stationary conditions are performed by first bringing the sample into a stationary condition and then at the initial time $t = 0$, suddenly turning off the temperature gradient, so that the sample gradually relaxes toward a homogeneous equilibrium state. Under these conditions non-equilibrium temperature fluctuations are not present, and the exponential decay of the correlation function is entirely determined by the relaxation of non-equilibrium concentration fluctuations. After a short transient with a duration of the order of $\tau _\textrm{T} \approx$ 100 s dictated by the thermal inertia of the sample cell, the system reaches an isothermal state, where the temperature profile is fully relaxed and the associated temperature NEFs are no longer present. The relaxation of the concentration profile requires a much longer time $\tau _\textrm{c}\approx h^2/(D_0\pi ^2)\simeq$ 900 s, during which time-dependent concentration NEFs are expected to be still detectable. The experiments described above have been repeated multiple times, starting from different initially imposed temperature differences $\varDelta T$ (4 K, 8 K, and 17 K). The cell is mounted on a support that can be tilted, so to achieve different values of the angle $\theta$ between the direction perpendicular to the cell plates and the acceleration of gravity and to mimic the small unavoidable misalignments present in experiments.

### Image acquisition and analysis

Each experiment consists of the acquisition, starting from the initial time $t=0$, of a sequence of $N=60,000$ images of the sample at a constant frame rate ${f_0}=30$ Hz. To visualize non-equilibrium fluctuations, images are acquired using an optical technique called dynamic shadowgraphy [10]. The sample is illuminated with a collimated, spatially coherent beam of light, which is partially scattered by fluctuations of the index of refraction determined by temperature and concentration fluctuations. As the transmitted beam propagates away from the fluid layer, it interferes with the scattered light and gives rise to an intensity pattern on the sensor. The distribution of this pattern reveals the non-uniformity of the local refractive index [54, 55], and the statistical analysis of the shadowgraph images allows characterizing both the static and dynamic structure factor of the sample. To apply this technique, we employ the optical setup shown in Fig. 1a. The light passing through the sample and generating the images is emitted by a superluminous diode (Superlum, SLD-MS-261-MP2-SM) with a wavelength of $\lambda = (675 \pm 13)$ nm. The diode is placed on the focal plane of an achromatic doublet with a focal length of $f = 200$ mm, which collimates the beam. Dynamic shadowgraph images were acquired with a scientific sCMOS camera (PCO.panda) positioned at distance $z = 45$ cm from the sample cell.

The total duration of the acquisition $t_\textrm{max}=N/{f_0}=2000$ s exceeds the characteristic diffusion time across the sample cell $\tau _\textrm{c}\simeq 900$ s. The image resolution, upon $4\times 4$ binning, is $512\times 512$ pixels, while the effective pixel size, is $d_\textrm{eff}=26\,\upmu \text {m}$. The collected images are analyzed according to a Fourier domain-based differential algorithm, often referred to as differential dynamic analysis (DDA) [10]. In the context of optical microscopy, the same approach is better known as differential dynamic microscopy (DDM) [23, 25]. A detailed description of the method can be found in Refs. [23, 25]. In brief, DDA is based on the calculation of the so-called image structure function:

1 $$\begin{aligned} D(\textbf{q},\varDelta t)=\langle |{\hat{I}}(\textbf{q},\varDelta t+t)-{\hat{I}}(\textbf{q},t)|^2\rangle _t, \end{aligned}$$

where ${\hat{I}}(\textbf{q},t)$ is the two-dimensional Fourier transform of the image intensity distribution $I(\textbf{x},t)$ collected at time *t*, and the symbol $\langle \cdot \rangle _t$ indicates a temporal average with a fixed time delay $\varDelta t$. Under suitable conditions, namely, if a linear space-invariant relation exists between image intensity and refractive index distribution within the sample [25], $D(\textbf{q},\varDelta t)$ encodes information on the relaxation dynamics and the mean squared amplitude of the corresponding Fourier mode, with wave vector $\textbf{q}$.

The analytical models used to extract such information will be discussed in more detail in the next section.

Under non-stationary conditions, we perform DDA at different stages during the relaxation of the concentration profile, considering time windows of amplitude *T* = 200 s, centered around $\bar{t} = [300, 450, 675, 1013, 1520, 1800]$ s. Accordingly, the first image to be considered in the analysis is the one collected at $t=200$ s, when the contribution of thermal NEFs, as well as possible artifacts due to mechanical displacements of parts of the setup induced by thermal expansion, is expected to be negligible.

## Modeling the effect of a uniform drift in the images

A typical shadowgraph image of the gradient cell under spatially coherent illumination exhibits a strong optical background, on top of which the subtle intensity modulations due to temperature and concentration fluctuations are superimposed (Fig. 1b). The background intensity distribution results from the superposition of the diffraction patterns of dust particles, scratches on the surfaces of lenses and confining plates, and impurities present along the optical path. In a typical shadowgraph experiment performed under stationary conditions, the optical background does not affect the measurement, as it is efficiently subtracted by the differential procedure.

In Fig. 1, we report representative image sequences and image structure functions obtained in experiments performed under stationary (c,d) and non-stationary (e,f) conditions, respectively. As can be appreciated from the figure, while in stationary conditions taking the difference between two images collected at different time points is very effective in removing all stray-light contributions, this is not the case for the non-stationary experiment. In this case, the image differences display a superimposed intensity pattern resembling the static background (see Fig. 1a), whose contrast increases with the time delay between the images, a behavior compatible with the presence of a rigid translation of the whole image.

To test this hypothesis, we introduce in this section a simple analytical model accounting for the effect of a slow, uniform drift in the images collected during a dynamic shadowgraph experiment. We show that such a drift introduces a spurious additive term in the image structure functions, similar to the one shown in Fig. 1f, whose amplitude and functional form can be calculated from the drift velocity and the spectrum of the image background. In the next sections, the validity of the model will be systematically tested against the experimental data, and the physical origin of the drift observed during the transient phase will be investigated.

Following Ref. [24], we write the image intensity distribution $I(\textbf{x},t)$, as the sum of three independent contributions

2 $$\begin{aligned} I(\textbf{x},t)=\delta I(\textbf{x},t)+I_0(\textbf{x})+I_N(\textbf{x},t), \end{aligned}$$

where $\delta I(\textbf{x},t)$ accounts for the intensity fluctuations generated by refractive index inhomogeneities within the sample, $I_0(\textbf{x})$ is the background intensity distribution, and the term $I_N(\textbf{x},t)$ accounts for the noise in the detection chain. We assume $I_N(\textbf{x},t)$ to have zero-mean $\langle I_N\rangle =0$ and to be delta-correlated in space and time $\langle I_N(\textbf{x}+\varDelta \textbf{x},t+\varDelta t)I_N(\textbf{x},t)\rangle =\langle I_N^2\rangle \delta (\varDelta \textbf{x})\delta (\varDelta t)$.

### Stationary conditions: no drift

We start considering the case where there is no drift in the images. Substituting Eq. 2 into Eq. 1, we obtain:

3 $$\begin{aligned} D(\textbf{q},\varDelta t)= & {} \langle |\delta {\hat{I}}(\textbf{q},\varDelta t+t)-\delta {\hat{I}}(\textbf{q},t)|^2\rangle _t+2\langle |{\hat{I}}_N(\textbf{q},t)|^2\rangle _t\nonumber \\= & {} A(\textbf{q})(1-\Re \{f(\textbf{q},\varDelta t)\})+B(\textbf{q}), \end{aligned}$$

where $A(\textbf{q})=2\langle |\delta {\hat{I}}(\textbf{q},t)|^2\rangle _t$ is proportional to the static scattering amplitude of the fluctuations [10], $B(\textbf{q})=2\langle |{\hat{I}}_N(\textbf{q},t)|^2\rangle _t$ accounts for the camera noise, the symbol $\Re \{\cdot \}$ stands for the real part, and $f(\textbf{q},\varDelta t)$ is the intermediate scattering function (ISF), which encodes the dynamics of the fluctuations [56]. If, as in the case of interest for this work, the dynamics is isotropic, one can also consider the azimuthally averaged image structure function $D(q,\varDelta t)=\langle D(\textbf{q},\varDelta t)\rangle _{q=\sqrt{q_x^2+q_y^2}}$. If the dynamics is characterized only by a single exponential decay, the image structure function takes the form:

4 $$\begin{aligned} D(q,\varDelta t)=A(q)\big (1-\text {e}^{-\varGamma (q)\varDelta t}\big )+B(q), \end{aligned}$$

where $\varGamma (q)$ is the relaxation rate of the process.

As shown in Fig. 1d, the normalized image structure functions $d(q,\varDelta t)=\frac{D(q,\varDelta t)-B(q)}{A(q)}$ under stationary conditions at large delay times saturates to a constant value, due to the decorrelation of the fluctuations.

### Non-stationary conditions: uniform drift

We consider now the effect of a rigid translation of the whole image with constant velocity $v_0$ during the experiment. Let us introduce the “moving” intensity distribution $I_M(\textbf{x},t)$ which, in terms of the stationary intensity distribution $I(\textbf{x},t)$, reads

$$\begin{aligned} I_M(\textbf{x},t)=I(\textbf{x}+\textbf{v}_0t,t). \end{aligned}$$

Taking a spatial Fourier transform of both sides, and applying the shift theorem, the above equation takes the form

5 $$\begin{aligned} {\hat{I}}_M(\textbf{q},t)=\text {e}^{-j\textbf{q}\cdot \textbf{v}_0t}{\hat{I}}(\textbf{q},t). \end{aligned}$$

We can now calculate the image structure function

$$\begin{aligned} D(\textbf{q},\varDelta t)=\langle |{\hat{I}}_M(\textbf{q},\varDelta t+t)-{\hat{I}}_M(\textbf{q},t)|^2\rangle _t, \end{aligned}$$

which, using the expression in Eq. 2, can be written as

6 $$\begin{aligned}{} & {} D(\textbf{q},\varDelta t)=|1-\text {e}^{-j\textbf{q}\cdot \textbf{v}_0\varDelta t}|^2|{\hat{I}}_0(\textbf{q})|^2\nonumber \\{} & {} +\langle |\text {e}^{-j\textbf{q}\cdot \textbf{v}_0\varDelta t}\delta {\hat{I}}(\textbf{q},t+\varDelta t)-\delta {\hat{I}}(\textbf{q},t)|^2\rangle _t+2\langle |{\hat{I}}_N^2(\mathbf {q,t)}\rangle _t,\nonumber \\ \end{aligned}$$

or, using the notation of Eq. 3

7 $$\begin{aligned} D(\textbf{q},\varDelta t)= & {} 2[1-\cos {(\textbf{q} \cdot \textbf{v}_0 \varDelta t)}]|{\hat{I}}_0(\textbf{q})|^2\nonumber \\{} & {} +A(\textbf{q})[1-\cos {(\textbf{q} \cdot \textbf{v}_0 \varDelta t)}\Re \{f(\textbf{q},\varDelta t)\}\nonumber \\{} & {} -\sin {(\textbf{q} \cdot \textbf{v}_0 \varDelta t)}\Im \{f(\textbf{q},\varDelta t)\}]+B(\textbf{q}),\nonumber \\ \end{aligned}$$

where $\Im \{\cdot \}$ stands for the imaginary part. Assuming that the displacement $\varDelta \textbf{x}$ of images during the time interval $\varDelta t$ is small $|\varDelta \textbf{x}|=|\textbf{v}_0|\varDelta t \ll 1/q$, we can expand the cosine and sine functions up to the second order in $|\textbf{v}_0|q\varDelta t$

$$\begin{aligned}{} & {} \cos {(\textbf{q} \cdot \textbf{v}_0 \varDelta t)}\simeq 1+\frac{1}{2}(\textbf{q } \cdot \textbf{v}_0 \varDelta t)^2,\nonumber \\{} & {} \sin {(\textbf{q} \cdot \textbf{v}_0 \varDelta t)}\simeq \textbf{q } \cdot \textbf{v}_0 \varDelta t. \end{aligned}$$

Substitution into Eq. 7 yields

8 $$\begin{aligned}{} & {} D(\textbf{q},\varDelta t)\simeq A(\textbf{q})(1-\Re \{f(\textbf{q},\varDelta t)\}\nonumber \\{} & {} \quad -\textbf{q } \cdot \textbf{v}_0 \varDelta t \; \Im \{f(\textbf{q},\varDelta t)\})+\alpha (\textbf{q})\varDelta t^2+B(\textbf{q}), \end{aligned}$$

where $\alpha (\textbf{q})$is related to the drift velocity $\textbf{v}_0$ through the relationship

9 $$\begin{aligned} \alpha (\textbf{q})=(\textbf{q}\cdot \textbf{v}_0)^2|{\hat{I}}_0(\textbf{q})|^2. \end{aligned}$$

If the genuine dynamics is isotropic and characterized by a single exponential relaxation with rate $\varGamma (q)$, the azimuthal average of Eq. 8 takes the form

10 $$\begin{aligned} D(q,\varDelta t)\simeq A(q)\big (1-\text {e}^{-\varGamma (q)\varDelta t}\big )+\alpha (q)\varDelta t^2+B(q), \end{aligned}$$

with

11 $$\begin{aligned} \alpha (q)=\frac{1}{2}q^2v_0^2|{\hat{I}}_0(q)|^2. \end{aligned}$$

In obtaining Eqs. 10 and 11, we have made use of the identities $\langle \textbf{q}\cdot \textbf{v}_0\rangle _{q=\sqrt{q_x^2+q_y^2}}=0$, and $\langle (\textbf{q}\cdot \textbf{v}_0)^2\rangle _{q=\sqrt{q_x^2+q_y^2}}=1/2q^2v_0^2$. Equations 10, 11 represent the main result of this section, as they show how a slow global drift in the images gives rise to an additive term, quadratic in $\varDelta t$, in the image structure function $D(q,\varDelta t)$, whose *q*-dependent amplitude results from a combination of the drift velocity and the spectrum of the background intensity distribution.

It is worth mentioning that, besides explicitly including a drift term in the image structure function as done in Eq. 10, other strategies could be attempted to deal with a drift in the data. For example, at least in principle, the effect of a global drift could be removed by applying a registration algorithm (see, e.g., [57]) to the images before performing DDA. Unfortunately, this approach does not represent a viable option in the present case for two main reasons. On the one hand, as discussed in detail in the following section, the drifts observed in our experiment are very slow (corresponding to displacements between consecutive frames of the order or $10^{-3}$ pixels). As such, they are not easy to capture by the cross-correlation algorithms used by most registration routines and are even more challenging to compensate for with the required precision. Moreover, not all the features present in the images participate in the drift. For example, the dark spots due to dust particles present on the camera sensor’s surface are perfectly static. This situation, corresponding to the simultaneous presence of different features in *relative* motion, cannot be dealt with by any rigid registration procedure. This feature, combined with the fact that the optical background has much stronger contrast than the fluctuating signal, also prevents the use of other variants of DDA like, for example, far-field DDM [58] which should be intrinsically less sensitive to drifts.

A further alternative strategy could be, instead of performing the azimuthal average of the image structure function, to consider only $\textbf{q}$-vectors orthogonal to the drift direction, i.e., such that $\textbf{q}\cdot \textbf{v}_0=0$, as this would automatically eliminate all drift-related terms in Eq. 8 (see, e.g., Ref. [59]). Unfortunately, besides introducing an additional step in the analysis (the identification of the drift direction, which is not known a priori), this procedure would also lead to a dramatic degradation of the signal-to-noise ratio in the image structure functions, due to the poorer statistics. Given the extremely low signals typical of these experiments, this would make practically impossible to extract any meaningful information on the sample dynamics.

**Fig. 2:**
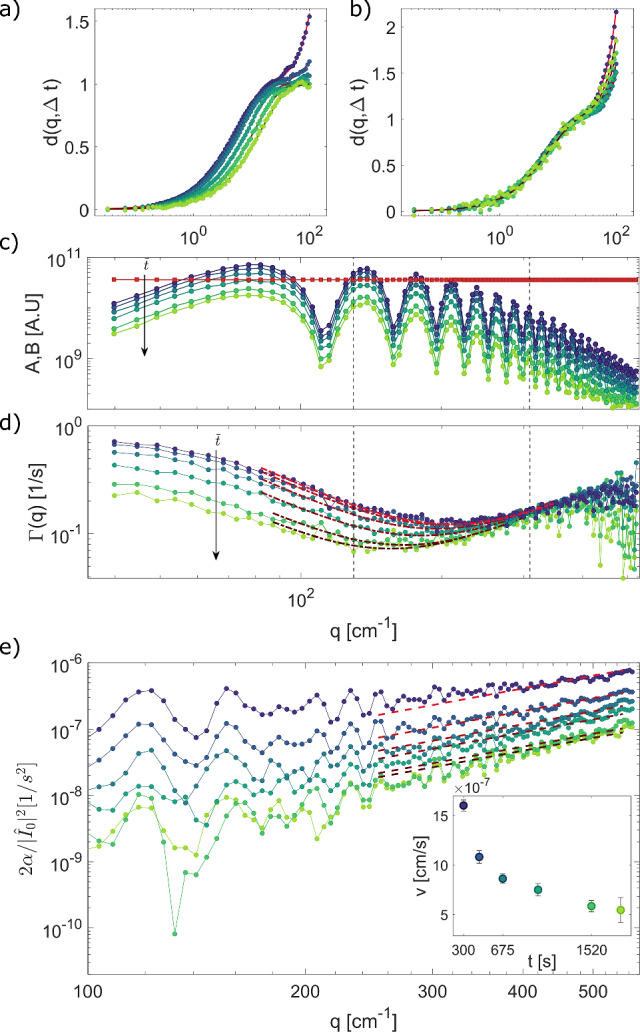
Differential analysis: **a**, **b** Representative normalized image structure functions of non-equilibrium concentration fluctuations during a transient diffusion process at different $\bar{t}$ ranging from $\bar{t}=300$ s (blue) to $\bar{t}=1800$ s (green), for $q=1.3\cdot 10^2$ cm$^{-1}$ **a** and $q=3.9\cdot 10^2$ cm$^{-1}$ **b**. The continuous lines are the best fitting curve assuming as a model Eq. 10. Time evolution of the mean squared amplitude **c** and relaxation rates (**d**) of non-equilibrium fluctuations during the approach to steady state. In (**c**), red-squared symbols represent the noise contribution *B*(*q*), which is identical at all the times $\bar{t}$. *A*(*q*) exhibits oscillations in *q*, due to the modulation of the typical shadowgraph transfer function [54]. In **d**, the dot-dashed lines correspond to the best fitting curve to $\varGamma (q)$ with a model $\varGamma (q)=D_{0}q^2\big [1+\big ( \frac{q_{ro}}{q} \big )^4\big ]$ [44]. The black vertical dashed lines correspond to the *q*-values of **a**, **b**, respectively. **e** Ratio between $\alpha (q)$ obtained from the fit to the ISFs with Eq. 10 and the power spectrum of the static image intensity optical background $|{\hat{I}}_0(q)|^2$. The red dashed lines represent the best fitting curve with a quadratic model. The fit has been performed over wave vectors in the range [2.5–5.5]$\,\cdot \,10^2$ cm$^{-1}$. Inset: drift velocity estimated from the fit plotted as a function of time

## Results and discussion

In the previous section, we obtained an analytical expression for the image structure function in the presence of a uniform drift with constant velocity $\textbf{v}_0$ in the images. In the following, we show that this model accurately captures the dynamical behavior observed during a typical experiment in non-stationary conditions. As described in Sect. 2, we consider experiments in which the system undergoes a transition from a stationary non-equilibrium condition (characterized by the presence of a steady temperature and concentration gradient across the sample) toward an equilibrium, homogeneous, one. After the temperature gradient is turned off, the temperature inside the sample becomes homogeneous nearly instantaneously, because the relaxation time of the macroscopic concentration profile is much larger than the thermal equilibration time. For this reason, the system is always gravitationally stable and we can rule out the presence of thermohaline convection.

Representative normalized image structure functions obtained at different times $\bar{t}$ after suddenly switching to zero the initial temperature difference $\varDelta T= 17$ K are reported in Fig. 2 a-b, for two different wave vectors $q=1.3\cdot 10^2$ cm$^{-1}$ (a) and $3.9\cdot 10^2$ cm$^{-1}$ (b). Continuous lines correspond to the best fitting curves using the model in Eq. 10. From the fitting procedure, we can simultaneously determine, for each time $\bar{t}$, the amplitude *A*(*q*) and the relaxation rate $\varGamma (q)$ of the solutal NEFs (Fig. 2c,d), the noise *B*(*q*) (Fig 2c), as well as the drift-related term $\alpha (q)$ (Fig. 2e).

A thorough discussion of the temporal evolution of the statics and the dynamics of NEFs during the transient phase leading to equilibrium is beyond the scope of this technical paper and will be addressed in future work. Here, we merely note that the *q*-dependent relaxation rate $\varGamma (q)$ (Fig. 2d)) is well captured at all times $\bar{t}$ by the theoretical expression [44]

12 $$\begin{aligned} \varGamma (q)=D_0q^2\bigg [1+\bigg (\frac{q_{ro}}{q}\bigg )^4\bigg ], \end{aligned}$$

which holds when a constant concentration gradient $\nabla c$ is present across the sample. In the above expression, $q_\textrm{ro}=(\chi g \nabla c / \nu D_0)^{1/4}$ is the so-called roll-off wave vector, which describes the effect of gravity on NEFs [6, 22, 31], where $\chi$ is the solutal expansion coefficient. Fitting Eq. 12 to the data enables estimating the mass diffusion coefficient, which is almost time-independent $D=(1.45\pm 0.04)\cdot 10^{-6}$ cm$^{2}$/s, as well as the roll-off wave vector $q_{ro}$, which displays over time a progressive shift toward lower and lower values, as predicted by theory.

According to Eq. 11, we expect the ratio ${2\alpha (q)}/{|{\hat{I}}_0(q)|^2}$ to scale as $q^2$, the proportionality constant being the squared modulus $v_0^2$ of the drift velocity. As it can be appreciated from Fig. 2e, the ratio displays a rather clean quadratic dependence on *q*, at least in the wave vector range $q \in [2.5-5.3]\cdot 10^2$ cm$^{-1}$, which corresponds to the regime where the contribution of the drift-related term to the image structure function is particularly relevant. Exploiting Eq. 11, we can thus evaluate the global drift velocity by fitting a quadratic model to ${2\alpha (q)}/{|{\hat{I}}_0(q)|^2}$. The static background contribution $|{\hat{I}}_0(q)|^2$ is obtained as the azimuthal average of the power spectrum of $\langle I(\textbf{x},t) \rangle _t$, where the temporal average is performed over the considered time window (see also Eq. 2).

The absolute value $v_0$ of the estimated drift velocity decreases over time while approaching the stationary regime (inset of Fig. 2e). Taken together, the results show that the simple model introduced in the previous section provides a fully consistent formal description of the experimental structure functions of non-equilibrium fluctuations during a transient diffusion process, and it is effective in decoupling the genuine dynamics of NEFs and the spurious drift associated with the rigid translation of the shadowgraph images at all stages of the experiment.

**Fig. 3:**
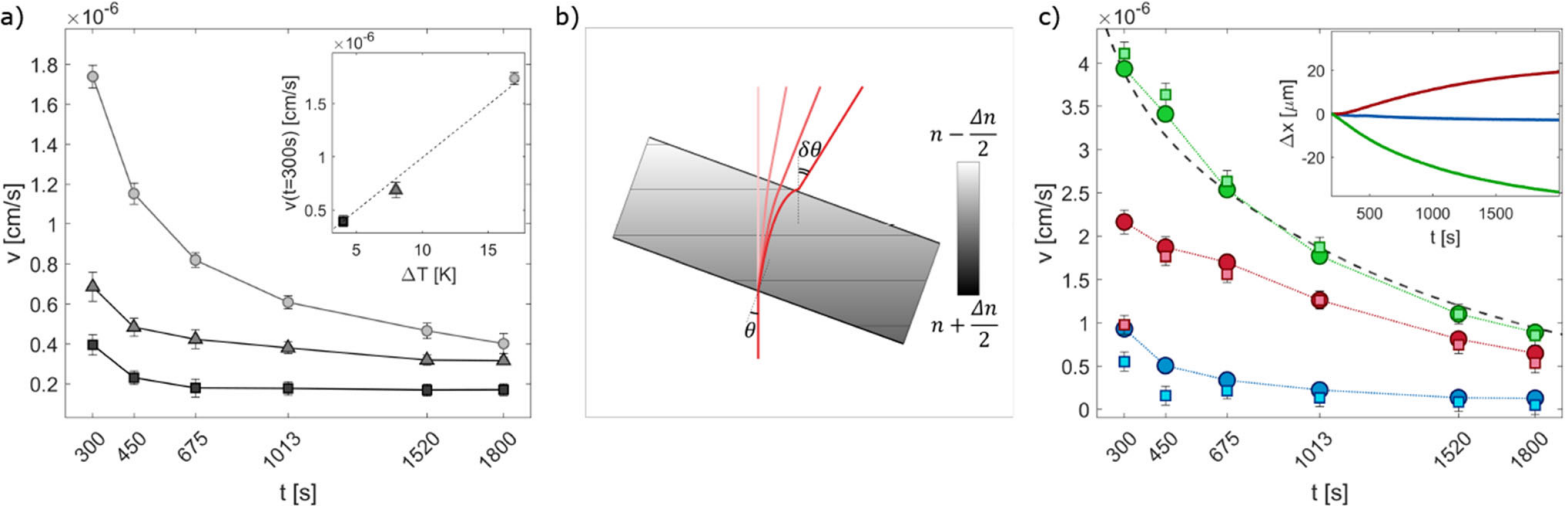
Drift Velocity: results for the drift velocity *v*, estimated from Eq. 11. **a** Velocity for the same angle of inclination and different temperature gradients $\varDelta T=4$ K ( black squares), 8 K (dark gray triangles) and 17 K (light gray circles). Inset: velocity estimated at time $t=300$ s as a function of the temperature gradient. **b** Schematic illustration of the effect of the cell tilt on the optical beam displacement. The sample cell is tilted by an angle $\theta$ with respect to gravity. Without loss of generality, the average refractive index *n* of the sample is assumed to be the same as the surrounding medium. At the beginning of the experiment, the sample is in a stratified state, characterized by a uniform refractive index gradient parallel to gravity, with a higher (lower) refractive index near the top (bottom) plate of the gradient cell. The horizontal gray lines correspond to isorefractive surfaces. In this case, according to Snell’s law, the presence of a component of the refractive index gradient perpendicular to the beam determines a deflection by an angle $\delta \theta \simeq \varDelta n \theta$ with respect to the incident direction (dark red line), where $\varDelta n$ is the refractive index difference across the cell. As the concentration gradient progressively relaxes by diffusion, the angular deflection becomes less and less pronounced until it vanishes when a homogeneous state is reached (light red lines). **c** Drift velocity for different angles of the gradient cell with respect to gravity, nominally $\theta =-1.3\cdot 10^{-2}$ rad (green), $+1.3\cdot 10^{-2}$ rad (red) and 0 rad (blue). Circles represent the velocity estimated from the differential dynamic analysis and squares stand for the velocity computed by the real-space tracking of the global displacement. The dashed line represents, up to a multiplicative factor, the time derivative of the concentration difference across the sample. Inset: real-space estimated global displacement in time for the three different inclination angles, plotted with the same color scheme

A problem that remains open is the physical origin of the drift observed during the relaxation of the concentration profile. A useful indication to address this point comes from the inspection of the temporal evolution of the estimated drift velocity (see inset of Fig. 2e), which displays a marked decrease over time while approaching the stationary regime. This observation, combined with the fact that we do not observe any drift under stationary conditions, suggests that the drift could be directly related to the progressive relaxation of the concentration gradient across the cell. To test this hypothesis, we repeated the above-described experiment for different values of the temperature difference $\varDelta T$ between the plates of the gradient cell, and thus of the initial concentration gradient $\nabla c=S_T c (1-c) \varDelta T/h$. As can be observed in Fig. 3a, the drift velocity clearly depends on the imposed temperature difference. Plotting the velocity measured at a fixed time point $\bar{t}=300$ s as a function of $\varDelta T$ reveals a nice linear dependence (inset of Fig. 3a), indicating that the drift velocity is directly proportional to the amplitude of the concentration gradient across the sample.

A possible explanation for this behavior can be provided as follows. In a liquid mixture subject to a thermal gradient $\varDelta T/h$, a concentration gradient builds up, due to the Soret effect. Neglecting gravity, the concentration gradient would be perfectly parallel to the imposed temperature gradient and thus orthogonal to the confining plates. On the other hand, in the presence of gravity, assuming that the sample cell is slightly inclined with respect to the horizontal direction, the stationary state of the mixture would entail the presence of a non-convective flow parallel to the confining plates, with a velocity field that deforms the concentration profile leading to isoconcentration surfaces perpendicular to gravity [50, 60, 61]. This situation is schematically represented in Fig. 3b, where we have assumed without loss of generality that the average index of refraction of the sample is equal to that of the surrounding medium. According to the discussion above, in the presence of a steady temperature difference across the cell, a vertical concentration gradient is present. After switching off the temperature difference, the mixture rapidly reaches an isothermal state. In this condition, the refractive index within the fluid only depends on the local concentration. In particular, the refractive index drop across the cell is given by $\varDelta n=\frac{\partial n}{\partial c} \varDelta c$, where $\varDelta c$ is the concentration difference between the plates. The fact that the confining plates are inclined by an angle $\theta$ with respect to gravity, while the concentration gradient is parallel to it, determines the presence of a component of $\varDelta n$ parallel to the plates. The stratified cell thus behaves like a prism, which imposes an angular deflection $\delta \theta \simeq \theta \varDelta n$ to a collimated beam impinging on it (Fig. 3b, dark red line). Over time, the concentration difference between the plates progressively decreases, and so does the refractive index drop across the cell, leading to a change in the angular deflection of the transmitted beam (Fig. 3b, light red lines). In our collection geometry (see Fig. 1a), any small angular change in the propagation direction of the transmitted beam leads to a proportional displacement of the intensity distribution on the sensor plane. We can thus write the relation between the velocity associated with the time-dependent linear displacement of the transmitted beam on the sensor plane and the rate of change of $\varDelta c$ as

13 $$\begin{aligned} v_0 \propto \theta \frac{\partial n}{\partial c} \frac{\text {d}}{\text {d}t}\varDelta c. \end{aligned}$$

**Fig. 4:**
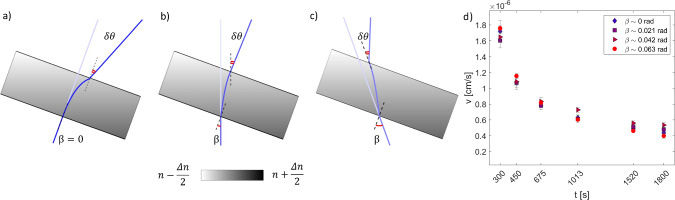
Drift Velocity for different angles of incidence: **a**–**c** schematic illustration of the effect of variable angle of incidence $\beta$ of the main beam on the sample cell. If, as in the case shown in the figures, the refractive index gradient and the inclination of the cell with respect to gravity are kept constant, the deflection $\delta \theta$ is not expected to depend on $\beta$. **d** Accordingly, different experiments, corresponding to different values of $\beta$, provide consistent results for the time evolution of the drift velocity

To validate this picture, we performed additional experiments, this time by carefully controlling the tilt angle $\theta$ of the cell, which is rotated around a horizontal axis. Nominally, we used $\theta =1.3\cdot 10^{-2}$ rad, 0 rad and $-1.3\cdot 10^{-2}$ rad. The results are shown in Fig. 3c. We observe that for $\theta =\pm 1.3\cdot 10^{-2}$ rad (green and red markers), the velocity is significantly larger than for $\theta =0$ rad (blue markers). We attribute the discrepancy between the two symmetric angles $\theta =\pm 1.3\cdot 10^{-2}$ rad to a systematic error in the alignment of the sample cell, which we estimate to be $\varDelta \theta =3.5\cdot 10^{-3}$ rad. In addition, the time dependence of the drift velocity is in excellent agreement with the time derivative of the concentration difference $\varDelta c$ across the sample (dashed black line in Fig. 3c—numerically calculated by solving the diffusion equation with suitable boundary and initial conditions [62]). To check the accuracy of these results, we performed also a real-space analysis by tracking the global displacement. This is done by using a customized cross-correlation-based registration algorithm based on the Image-J *Stack-Reg* plugin, which returns the transformation matrices of the registration [57, 63]. The cross-correlation analysis is performed on a sub-sequence obtained by keeping one image every thirty. In this way, the typical displacement between consecutive images becomes large enough (of the order of $10^{-2}$ pixels) to be reliably measured. We compute the global displacement from the transformation matrices, and then the mean velocity on the same times $\bar{t}$ considered in the differential analysis. The obtained results, shown as square symbols in Fig. 3c, are in very good agreement with the velocities estimated with the differential analysis (circles). We also checked that, upon changing the sign of the tilt angle, the image drifts in the opposite direction (inset of Fig. 3c), providing further compelling evidence that the velocity is strongly dependent on the tangential component of the gravity parallel to the fluid layers. To complete the systematic investigation of the experimental parameters that could determine a drift of the images, we analyzed the dependence of the drift velocity on the inclination angle $\beta$ of the main beam with respect to the normal to the optical sapphire windows. We did this by keeping fixed the angle $\theta$ between gravity and the normal to the optical windows and displacing the light source perpendicularly to the optical axis to change the inclination of the illumination beam impinging on the sample cell. The experimental results shown in Fig. 4 show that the deflection of the beam is not affected by $\beta$, and this is confirmed by simulations of the ray tracing inside the cell. This result provides further evidence that the only physical effect responsible for the drift of the images is the alignment of the layer of sample with respect to gravity.

## Conclusion

In this work, we have addressed an experimental problem experienced in the investigation with optical means of non-equilibrium fluctuations in fluid mixtures under non-stationary conditions, namely the presence of a time-dependent quadratic contribution in the measured structure functions. Until now, the presence of this contribution has been addressed empirically, but the significant investment made for the development of the Giant Fluctuations and TechNES projects of ESA has made a full understanding of its origin a priority for the analysis of the huge amount of data that will be generated during these space missions. We have shown that this contribution is due to a progressive deflection of the probe beam, leading to a global translation in the intensity distribution on the sensor plane. This is caused by the evolving concentration profile within the cell, the drift velocity being directly proportional to the time derivative of the concentration difference across the cell. This effect is particularly relevant when the sample cell is tilted with respect to gravity, while it is minimized when it is almost perfectly horizontal. We have introduced an analytical model that, fitted to the experimental data, enables disentangling the effects of the drift and the genuine dynamics of the system, which can be thus reliably reconstructed. Our results, by providing practical indications on the design and the fine-tuning of the experimental setup, as well as analytical tools to correctly interpret the data, will enable the investigation of the largely unexplored domain of NEFs in non-stationary conditions.

Moreover, the simple analysis scheme proposed in this work could be relevant in a wider range of applications involving the use of Fourier domain-based quantitative imaging methods [64]. Indeed, the presence of intrinsic or spurious drifts that superimpose the signal of interest is a widespread feature, for example, in rheomicroscopy experiments [59, 65, 66], in the presence of advective flows [67], or collective directed migration [68]. We expect our methodology to be particularly effective in the presence of slow drifts involving a strong optical background, a regime where the use of alternative approaches requiring the precise identification of the drift direction or its accurate frame-by-frame compensation can be challenging.
